# Scan and share to register outpatients, India

**DOI:** 10.2471/BLT.24.292828

**Published:** 2024-12-03

**Authors:** Sutapa B Neogi, Vikram Pagaria, Sharbari Dutta, Nishikant Bele, Prateeksha Yadav, Ratika Samtani

**Affiliations:** a International Institute of Health Management Research, Phase 2, Plot No 3, Sector 18A, Dwarka, New Delhi, 10075, India.; b National Health Authority, Janpath, Connaught Place, New Delhi,110001, India.

## Abstract

**Problem:**

To address the long waiting times patients incur when visiting outpatient departments in India.

**Approach:**

In 2022, the National Health Authority in India developed a paperless service, called Scan and Share, leveraging mobile technology and QR (quick-response) codes to streamline outpatient department appointments. Patients can use a mobile application (app) to scan QR codes at health facilities, generating tokens linked to registration counters. The service integrates patients’ demographic data from their Ayushman Bharat Health Account with the facilities’ health information systems. Collaboration with government bodies, health workers and patient advocacy groups ensured compliance with data protection regulations. For developers, the National Health Authority published detailed technical specifications outlining standards for app functionality, security and interoperability on the Ayushman Bharat Digital Mission platform.

**Local setting:**

Launched in 2021, Ayushman Bharat Digital Mission in India aims to digitize health care, creating a shared digital infrastructure for affordable and accessible care.

**Relevant changes:**

As at 19 October 2024, the Scan and Share service operates in 16 939 health-care facilities across 35 states and territories. The service has reduced waiting times from 1 hour to 2–5 minutes. Out of 56 801 213 tokens generated, 714 014 (1.3%) were issued in the private sector. There are currently 2074 active developers, with 1545 from the private sector.

**Lessons learnt:**

The Scan and Share service is transforming health-care delivery by reducing outpatient department queues, waiting times and errors. High-performing states can provide valuable insights for states with low service adoption.

## Résumé

**Problème:**

Remédier aux longs délais d’attente que subissent les patients lorsqu’ils se rendent dans les services de consultation ambulatoire en Inde.

**Approche:**

En 2022, l’Autorité nationale indienne de santé a mis au point un service sans papier, appelé Scan and Share, qui repose sur la technologie mobile et les codes QR afin de rationaliser les rendez-vous dans les services ambulatoires. Les patients peuvent utiliser une application mobile pour scanner les codes QR dans les établissements de soins de santé, ce qui génère des jetons liés aux guichets d’enregistrement. Ce service intègre aux systèmes d’information sanitaire des établissements les données démographiques des patients provenant de leur compte Ayushman Bharat Health Account (programme public d’assurance maladie). La collaboration avec les organismes publics, les professionnels de la santé et les groupes de défense des patients a permis de garantir le respect des réglementations de protection des données. Pour les développeurs, l’Autorité nationale indienne de santé a publié des spécifications techniques détaillées décrivant les normes de fonctionnalité, de sécurité et d’interopérabilité de l’application sur la plateforme de la mission numérique Ayushman Bharat.

**Environnement local:**

Lancée en 2021, la mission numérique Ayushman Bharat en Inde vise à numériser les soins de santé, en créant une infrastructure numérique partagée pour des soins abordables et accessibles.

**Changements significatifs:**

Au 19 octobre 2024, le service Scan and Share fonctionne dans 16 939 établissements de santé répartis dans 35 États et territoires. Le service a permis de réduire les temps d’attente de 1 heure à 2-5 minutes. Sur les 56 801 213 jetons générés, 714 014 (soit 1,3%) ont été émis dans le secteur privé. On compte actuellement 2074 développeurs actifs, dont 1545 proviennent du secteur privé.

**Leçons tirées:**

Le service Scan and Share transforme la prestation des soins de santé en réduisant les files d’attente, les délais et les erreurs dans les services ambulatoires. Les États les plus performants peuvent fournir des informations précieuses à ceux qui n’ont pas adopté ce service.

## Resumen

**Situación:**

Abordar los tiempos largos de espera que experimentan los pacientes cuando acuden a los servicios ambulatorios en la India.

**Enfoque:**

En 2022, la Autoridad Sanitaria Nacional de la India desarrolló un servicio sin papeles, llamado Scan and Share, que aprovecha la tecnología móvil y los códigos QR (de respuesta rápida) para agilizar las citas en los servicios ambulatorios. Los pacientes pueden utilizar una aplicación móvil para escanear códigos QR en los centros sanitarios, que generan fichas vinculadas a las ventanillas de registro. El servicio integra los datos demográficos de los pacientes de su cuenta Ayushman Bharat Health Account con los sistemas de información sanitaria de los centros. La colaboración con organismos gubernamentales, personal sanitario y grupos de defensa del paciente garantizó el cumplimiento de la normativa sobre protección de datos. Para los desarrolladores, la Autoridad Sanitaria Nacional ha publicado especificaciones técnicas detalladas que describen los estándares de funcionalidad, seguridad e interoperabilidad de las aplicaciones en la plataforma de la misión digital Ayushman Bharat.

**Marco regional:**

Lanzada en 2021, la misión digital Ayushman Bharat en la India tiene como objetivo digitalizar la atención sanitaria, al crear una infraestructura digital compartida para una atención asequible y accesible.

**Cambios importantes:**

A 19 de octubre de 2024, el servicio Scan and Share funciona en 16 939 centros sanitarios de 35 estados y territorios. El servicio ha reducido los tiempos de espera de una hora a 2-5 minutos. De las 56 801 213 fichas generadas, 714 014 (1,3%) se emitieron en el sector privado. En la actualidad, hay 2074 desarrolladores activos, de los que 1545 pertenecen al sector privado.

**Lecciones aprendidas:**

El servicio Scan and Share transforma la asistencia sanitaria al reducir las filas, los tiempos de espera y los errores en los servicios ambulatorios. Los Estados con buenos resultados pueden aportar información valiosa a los Estados con baja adopción de servicios.

## ملخص

**المشكلة:**

مواجهة أوقات الانتظار الطويلة التي يقضيها المرضى عند زيارة أقسام العيادات الخارجية في الهند.

**الأسلوب:**

في عام 2022، قامت الهيئة الوطنية للصحة في الهند بتطوير خدمة لا تعتمد على استخدام الورق، تسمى Scan and Share (المسح الضوئي والمشاركة)، تستفيد من تكنولوجيا الهاتف المحمول ورموز الاستجابة السريعة (QR) لتبسيط مواعيد أقسام العيادات الخارجية. يمكن للمرضى استخدام تطبيق للهاتف المحمول لمسح رموز الاستجابة السريعة في المرافق الصحية، وإنشاء رموز مميزة مرتبطة بمكاتب التسجيل. تدمج هذه الخدمة البيانات السكانية للمرضى من حساب Ayushman Bharat Health الخاص بهم، مع أنظمة المعلومات الصحية للمرافق. إن التعاون مع الهيئات الحكومية، والعاملين في القطاع الصحي، ومجموعات دعم المرضى، يضمن الامتثال لقواعد حماية البيانات. وبالنسبة للمطورين، فقد نشرت الهيئة الوطنية للصحة مواصفات فنية مفصلة تحدد المعايير القياسية لوظائف التطبيق، والأمان، والتشغيل البيني على منصة Ayushman Bharat Digital Mission.

**المواقع المحلية:**

تم إطلاق مهمة Ayushman Bharat الرقمية في الهند في عام 2021، وهي تهدف لرقمنة الرعاية الصحية، وإنشاء بنية تحتية رقمية مشتركة للرعاية، بحيث يمكن الوصول إليها بسهولة وبأسعار معقولة.

**التغيّرات ذات الصلة:**

اعتبارًا من 19 أكتوبر/تشرين أول عام 2024، تم تشغيل خدمة Scan and Share في 16939 منشأة للرعاية الصحية في 35 ولاية وإقليم. وقد قللت الخدمة أوقات الانتظار من ساعة واحدة إلى 2 إلى 5 دقائق. من بين 56801213 رمزًا مميزًا تم إنشاؤه، تم إصدار 714014 (%1.3) في القطاع الخاص. يوجد حاليًا 2074 مطورًا نشطًا، منهم 1545 من القطاع الخاص.

ا **لدروس المستفادة **تعمل خدمة Scan and Share على تحويل تقديم الرعاية الصحية، وذلك من خلال تقليل طوابير أقسام العيادات الخارجية، وأوقات الانتظار، والأخطاء. يمكن للولايات ذات الأداء العالي أن تقدم رؤى ذات قيمة للولايات ذات معدل الخدمة المنخفض.

## 摘要

**问题:**

旨在解决印度患者在门诊部就诊时候诊时间过长的问题。

**方法:**

2022 年，印度国家卫生局开发了一种名为“扫描与共享”的无纸化服务，可利用移动技术和快速反应码简化门诊部预约流程。患者在医疗机构处通过移动应用程序扫描快速反应码，即可生成与挂号柜台相链接的令牌。该服务从国家健康保护计划健康账户 (Ayushman Bharat Health Account) 中调取患者的人口统计数据，并将此类数据整合至医疗机构的健康信息系统中。与政府机构、卫生工作者和患者倡导组织进行了合作以确保所提供服务遵循数据保护条例。开发人员需注意的是，国家卫生局发布了详细的技术规范，概述了国家健康保护计划数字任务 (Ayushman Bharat Digital Mission) 平台上的应用程序功能、安全性和互操作性相关标准。

**当地状况:**

印度国家健康保护计划数字任务平台于 2021 年启动，旨在实现卫生保健数字化，创建共享数字基础设施以提供负担得起且可获得的护理服务。

**相关变化:**

截至 2024 年 10 月 19 日，“扫描与共享”服务已在 35 个邦和联邦属地的 16,939 家卫生保健机构中投入使用。该服务将候诊时间从 1 个小时缩短至了 2-5 分钟。在生成的 56,801,213 个令牌中，有 714,014 个 (1.3%) 是由私营部门发出的。目前共有 2,074 名活跃的开发人员，其中 1,545 名来自私营部门。

**经验教训:**

通过采用“扫描与共享”服务，可减少门诊部排队人数、缩短候诊时间和降低错误率，从而实现卫生保健服务变革。取得卓越成效的邦可为服务采用率较低的邦提供宝贵的见解。

## Резюме

**Проблема:**

Сократить время ожидания, с которым сталкиваются пациенты при посещении амбулаторных отделений в Индии.

**Подход:**

В 2022 году Национальное управление здравоохранения Индии разработало цифровую услугу под названием Scan and Share, используя мобильные технологии и QR-коды (коды быстрого отклика) для упрощения записи на прием в амбулаторное отделение. Для сканирования QR-кодов в медицинских учреждениях пациенты могут использовать мобильное приложение, которое генерирует токены, привязанные к стойкам регистрации. Сервис объединяет демографические данные пациентов из приложения Ayushman Bharat Health Account с системами информации медицинских учреждений. Соблюдение правил защиты данных обеспечивалось в результате совместной работы государственных органов, медицинских работников и групп защиты прав пациентов. Национальное управление здравоохранения опубликовало для разработчиков подробные технические спецификации, определяющие стандарты функциональности, безопасности и совместимости приложений на платформе Ayushman Bharat Digital Mission.

**Местные условия:**

Запущенный в 2021 году проект Ayushman Bharat Digital Mission в Индии направлен на цифровизацию сферы здравоохранения, создание общей цифровой инфраструктуры для оказания недорогой и доступной медицинской помощи.

**Осуществленные перемены:**

По состоянию на 19 октября 2024 года сервис Scan and Share работает в 16 939 медицинских учреждениях в 35 штатах и территориях. Благодаря этому сервису время ожидания сократилось с 1 часа до 2–5 минут. Из 56 801 213 сгенерированных токенов 714 014 (1,3%) были произведены в частном секторе. В настоящее время насчитывается 2074 активных разработчика, из них 1545 – в частном секторе.

**Выводы:**

Сервис Scan and Share меняет систему оказания медицинской помощи, сокращая очереди в амбулаторных отделениях, уменьшая время ожидания и количество ошибок. Штаты с высокими показателями могут предоставить ценную информацию для штатов с низким уровнем внедрения услуг.

## Introduction

A well-functioning health-care system delivers high-quality services to the population, ensuring it is accessible, affordable and sustainable over time.^1^ In India, a concern for patients visiting secondary and tertiary care hospitals is the long queues for registration, causing considerable delays, and often forcing patients to return the next day. These delays are primarily due to manual data entry, errors in such entries and insufficient staff. To address this problem, the National Health Authority in India introduced a paperless service, called Scan and Share, which leverages mobile technology and QR (quick-response) codes to streamline outpatient department appointments. Through this service, patients can scan a facility’s unique QR code to register at the outpatient department. With their consent, they can also allow health workers access to their health records stored in the facility's hospital management information system. Here we describe how the Scan and Share service seeks to simplify health-care access for citizens visiting outpatient departments.

## Local setting

In 2021, the Indian government launched the Ayushman Bharat Digital Mission to digitize all health-care services and build a common digital infrastructure for low-cost and seamless health-care service delivery in India.^2^ The mission, governed by the health ministry and implemented by the National Health Authority, is funded by the Indian government with support from both the public and private sector. 

The mission has implemented six building blocks: (i) Ayushman Bharat Health Account identifier (ID), a voluntarily generated 14-digit unique personal ID that can be used across health-care facilities; (ii) individual-controlled personal health record applications (apps) that enables patients to securely view, link and share their medical records; (iii) a health professional registry, which serves as a comprehensive repository of all health workers across modern and traditional medicine systems; (iv) a health facility registry, a comprehensive directory of health facilities across various medical systems; (v) a drug registry; and (vi) a unified health interface providing open protocols that create an interoperable network of digital health services between facilities for patient-centric services.^3^ The interface offers diverse health record apps that users can download to explore services and facilities, book appointments and pay for health-care services. The replicability of unified health interface resembles the unified payments interface, used for instant payment, which has been successfully adopted in India.^3^

Additionally, technologies such as barcodes, QR codes, blockchain technology and radio frequency identification tags are being used to enhance the digital health-care infrastructure. 

Patients’ health data are stored locally within each facility's system, ensuring compliance with data protection regulations and Ayushman Bharat Digital Mission protocols.

## Approach

To reduce errors and expediting registration in hospital outpatient departments, the National Health Authority created a Scan and Share service in 2022. This service enables paperless registration and, with user consent, the service also supports secure sharing and real-time access of health records.^4^

Throughout the project, the authority consulted with government bodies, health-care providers and advocacy groups, assessed health facility infrastructure readiness, ensured data protection compliance and collaborated with the health ministry for regulatory approvals. The authority collaborated with about 30 hospital management information system vendors for integrating QR code functionality into existing hospital systems. The Advisory Group of the Ayushman Bharat Digital Mission, comprised of digital health experts and policy-makers, ensured that the project aligned with national health goals and standards.

For designing, testing and implementing the Scan and Share service, the National Health Authority contracted public and private technical developers specializing in digital health and interoperability. For interested app developers, the authority developed and published detailed technical specifications outlining standards for personal health record apps functionality, security and interoperability on an open application programming interface framework on the Ayushman Bharat Digital Mission platform. This interface framework provided a sandbox environment, allowing developers to test their apps with simulated data to ensure compliance with the digital mission’s ecosystem. Each app underwent a certification process to meet digital mission standards for data privacy, encryption and user interface. Only certified apps were authorized for use, and standardized QR codes had to be unique, secure and compatible with various health facilities. 

Before and during implementation, the National Health Authority conducted structured training sessions at various levels, including state governments, chief medical officers and facility managers. Virtual training sessions on Scan and Share service are held monthly for doctors, nurses and data entry operators. To educate health workers and patients on the registration process and increase understanding of the service, the authority organized awareness campaigns, using instructional videos, posters and banners, and conducted various outreach activities. Top-performing states share their practices with other states through cross-learning sessions. At the health facilities, health workers regularly received technical support and doubt-clearing sessions from the authority.

Facility staff also received training on the hospital management information system, which is provided by a combination of government health authorities, system vendors and companies specializing in health-care software.

To ensure that all patients have access to the Scan and Share service, the authority created two options to register at the outpatient department, either with a smartphone or using an Ayushman Bharat Health Account card (Box 1). People using a smartphone access the service through a personal health record app linked to their Ayushman Bharat Health Account.^5^ When patients scan a health facility's unique QR code, their health account is accessed through the cloud services of the National Health Authority. Using the demographic data in the account, the app generates a token linked to the facility or to a specific counter, such as those for elderly patients.^5^ Patients without a smartphone can use their Ayushman Bharat Health Account card, which displays a QR code. This code can be scanned at the registration desk to retrieve their demographic data from the health account. Patients without a card can generate one at dedicated hospital kiosks.

Box 1 **Options for patients to scan the QR code**

**For patients with smartphones**

The patient scans a QR code at the outpatient department registration counter, which directs them to the Ayushman Bharat Digital Mission-enabled personal health record app on their smartphone. The profile-sharing page appears in the app, and after the patient provides consent and clicks on the share button, the details linked to their Ayushman Bharat Health Account are sent to the health facility’s health management information system, completing the express registration. A waiting number, a so-called token, is generated and displayed on the app, allowing patients to wait for the number to appear on the registration screen. The token is valid for 60 minutes. At the counter, patients are asked basic health questions and a consultation slip is generated before patients are directed to a specific department.

If the patient does not have a personal health record app, a list of compliant apps is displayed after scanning the QR code. The user can select any application to download, and they can also create an Ayushman Bharat Health Account ID if needed.

**Patient lacking access to a smartphone or other digital tools**

Patients can obtain an Ayushman Bharat Health Account card at dedicated hospital kiosks, where it is created using biometric or face authentication. The Ayushman Bharat Health Account card displays a patient-specific QR code, which the patient can scan at the registration counter, allowing the hospital's health management information system to access the patient’s demographic data. The patient then proceeds to the required department for consultation with their outpatient department slip.

App: application; QR: quick response.

To be able to provide the Scan and Share service, hospitals need a hospital management information system compliant with the Ayushman Bharat Digital Mission standards, dedicated counters, token display boards, announcement systems and QR codes for patients to register. The hospital management information system links with Ayushman Bharat Health Account through the unique ID for follow-up patients, assigns new unique health IDs to first-time users, and sets token expiration time. To be able to generate QR codes for registration, hospitals must register with the health facility registry. For health workers to access a patient’s health records after consent, they must register with the health professional registry.

The Scan and Share service was implemented using funds from the Ayushman Bharat Digital Mission budget, with a total cost of 16 billon Indian rupees (190 240 000 United States dollars) over five years. This funding covered the infrastructure setup, software deployment and operational expenses required to implement the system. The service is offered free of charge to patients and facilities.

## Relevant changes

The Scan and Share service has been adopted by 35 out of 36 States and Union Territories of India, and is of October 2024 operational across 16 939 health-care facilities. More than 50 million tokens have been generated, reducing waiting times from 1 hour to 2–5 minutes. An average of 220 000 individuals use the service daily.^6^ The number of tokens generated varies across different states, with government facilities contributing to a greater share. Out of 56 801 213 tokens generated, 714 014 (1.3%) were issued by the private sector. The number of Ayushman Bharat Health Account IDs created also varied across the country (Table 1). More than half of these accounts have been issued to individuals aged 19–45 years. Additionally, 410 million health records have been linked to health account IDs.

**Table 1:** Number of Ayushman Bharat Health Account created and Scan and Share tokens generated, India, 19 October 2024

State or union territory	Population	Health accounts created, no (%)	No. (%) of tokens generated		
			Total	Government facilities	Private facilities
Uttar Pradesh	238 875 000	112 046 330 (46.9)	9 593 365	9 548 865 (99.5)	44 500 (0.5)
Maharashtra	127 684 000	52 720 069 (41.3)	719 605	716 419 (99.6)	3 186 (0.4)
Gujarat	72 653 000	45 670 972 (62.9)	347 810	345 318 (99.3)	2 492 (0.7)
Madhya Pradesh	87 954 000	44 986 998 (51.1)	1 095 793	1 093 021 (99.7)	2 772 (0.3)
Andhra Pradesh	53 402 000	41 432 145 (77.6)	5 669 164	5 668 903 (100.0)	261 (0.0)
West Bengal	99 723 000	35 478 690 (35.6)	186 538	186 504 (100.0)	34 (0.0)
Bihar	129 205 000	38 283 076 (29.6)	1 142 096	1 141 706 (100.0)	390 (0.0)
Rajasthan	82 188 000	34 688 959 (42.2)	74 695	74 667 (100.0)	28 (0.0)
Karnataka	68 256 000	28 351 246 (41.5)	4 103 190	4 091 793 (99.7)	11 397 (0.3)
Odisha	46 663 000	26 162 859 (56.1)	474 358	473 609 (99.8)	749 (0.2)
Chhattisgarh	30 638 000	21 296 619 (69.5)	680 244	680 136 (100.0)	108 (0.0)
Telangana	38 317 000	19 626 176 (51.0)	192 087	191 851 (99.9)	236 (0.1)
Assam	36 159 000	18 177 557 (50.3)	354 285	354 244 (100.0)	41 (0.0)
Kerala	35 967 000	17 622 631 (49.0)	1 243	1 219 (98.1)	24 (1.9)
Jharkhand	40 129 000	13 801 170 (34.4)	91 946	91 838 (99.9)	108 (0.1)
Haryana	30 694 000	13 991 898 (45.6)	224 163	20 (0.0)	224 143 (100.0)
Punjab	30 992 000	12 949 207 (41.8)	129 093	129 026 (99.9)	67 (0.1)
Tamil Nadu	77 165 000	12 418 244 (16.1)	151	101 (66.9)	50 (33.1)
Jammu And Kashmir	13 733 000	8 843 954 (64.4)	3 789 983	3 789 953 (100.0)	30 (0.0)
Delhi	21 884 000	8 306 527 (38.0)	2 139 972	2 139 400 (100.0)	572 (0.0)
Uttarakhand	11 795 000	6 740 933 (57.2)	214 717	214 697 (100.0)	20 (0.0)
Himachal Pradesh	7 518 000	5 917 464 (78.7)	367	365 (99.5)	2 (0.5)
Tripura	4 194 000	1 739 843 (41.5)	130 255	119 412 (91.7)	10 843 (8.3)
Puducherry	1 695 000	1 120 109 (66.1)	5 755	5 749 (99.9)	6 (0.1)
Meghalaya	3 387 000	1 081 796 (31.9)	23 784	23 784 (100.0)	0 (0.0)
Manipur	3 260 000	891 325 (27.3)	10 347	10 286 (99.4)	61 (0.6)
Goa	1 585 000	841 942 (53.1)	2 087	2 071 (99.2)	16 (0.8)
Chandigarh	1 247 000	698 574 (56.0)	102 339	102 334 (100.0)	5 (0.0)
Dadra and Nagar Haveli & Daman and Diu	1 387 000	683 275 (49.3)	279	279 (100.0)	0 (0.0)
Nagaland	2 258 000	677 180 (30.0)	NA	NA	NA
Mizoram	1 252 000	638 527 (51.0)	4	1 (25.0)	3 (75.0)
Sikkim	697 000	414 008 (59.4)	5 264	5 264 (100.0)	0 (0.0)
Arunachal Pradesh	1 580 000	357 539 (22.6)	10 211	10 211 (100.0)	0 (0.0)

NA: not available.

Several rounds of training have been held, covering health account ID creation, record linking, data privacy, consent management and system integration to ensure secure, interoperable health data management. As of November 2024, 204 government and private partners are creating Ayushman Bharat Health Account IDs, with 126 of them (93 from the private sector) linking patients’ health records to their health account IDs.

Downloaded state-wise data up to 19 October 2024, from the Ayushman Bharat Digital Mission public dashboard,^7^ showed that 341 973 health facilities were registered in the health facility registry, with 56.5% (193 348) being public facilities. Of the 486 879 health workers registered in the health professional registry, 40.4% (196 861) are doctors, 71.0% (345 684) from the government sector and 29.0% (141 195) from the private sector.

There are currently 2074 active developers in the sandbox environment, with 1545 from the private sector. Among 131 partners that have successful integrated with the digital health ecosystem, 111 (84.7%) are from the private sector.

## Lessons learnt

The Scan and Share service is transforming health-care delivery in India by reducing outpatient department registration queues and waiting times, benefiting vulnerable groups, and addressing inefficiencies and errors in manual data entry (Box 2). The open, standardized and secure interface framework, combined with the service’s interoperability, scalability and user-centric design have facilitated widespread adoption and integration across the health system. These features also support other QR code-enabled services, such as Scan and Send, and Scan and Pay, allowing patients to directly pay for laboratory tests and medications through the app. Therefore, the National Health Authority is now expanding its implementation to pharmacies and laboratories.

Box 2 **Summary of main lessons learnt**

- The Scan and Share service reduced waiting times for outpatient department registration
- By integrating the service into hospital management systems, health-care facilities experienced reduced manual data entry errors and lowered administrative burdens
- Challenges in limited digital infrastructure, unreliable internet connectivity and training needs are addressed with a financial incentive scheme

To boost adoption in states with low uptake, digital literacy, access to digital devices and health information systems, internet connectivity and infrastructure needs to be addressed, alongside targeted training for health workers and patients. Valuable insights and best practices from high-performing states can guide policy-makers in accelerating implementation and facilitating state-level adoption. Other challenges include limited private sector adoption, delays in policy implementation, fragmented information streams from multiple digital platforms and interoperability issues between the health information system and the sandbox interface. The National Health Authority is addressing the limited digital infrastructure, unreliable internet connectivity and training needs by offering financial incentives through the Digital Health Incentive Scheme.

India’s vast population, federal structure and cultural diversity present unique challenges to health-care provision. However, recent strides in digitally transforming the health sector have laid a foundation for continued growth and digital transformation.
